# Recent Advances in Hydrogels for the Diagnosis and Treatment of Dry Eye Disease

**DOI:** 10.3390/gels8120816

**Published:** 2022-12-11

**Authors:** Qiaoqiao Li, Yifeng Cao, Ping Wang

**Affiliations:** 1 College of Pharmaceutical Science, Zhejiang University of Technology, Hangzhou 310014, China; 2 Institute of Electronic Chemicals, Institute of Zhejiang University—Quzhou, Quzhou 324000, China; 3 Department of Chemical and Biological Engineering, Zhejiang University, Hangzhou 310058, China

**Keywords:** hydrogels, dry eye disease, diagnosis and treatment, biosensor, controlled release

## Abstract

Dry eye disease (DED) is the most common clinical ocular surface disease. Given its multifactorial etiology, no consensus has been reached on the diagnosis criteria for dry eye disease. Topical drug administration remains the mainstay of treatment but is limited to the rapid clearance from the eye surface. To address these problems, hydrogel-based materials were designed to detect biomarkers or act as drug delivery systems by taking advantage of their good biocompatibility, excellent physical and mechanical properties, and long-term implant stability. Biosensors prepared using biocompatible hydrogels can be sensitive in diagnosing DED, and the designed hydrogels can also improve the drug bioavailability and retention time for more effective and long-term treatment. This review summarizes recent advances in the use of hydrogels for diagnosing and treating dry eye, aiming to provide a novel reference for the eventual clinical translation of hydrogels in the context of dry eye disease.

## 1. Introduction

Dry eye disease (DED) is a complex ocular surface disorder occurring when the tears cannot provide sufficient lubrication for the eyes, which causes eye discomfort, damage to the eye tissues, or even significant loss of vision [1]. The prevalence of dry eye has been established to range from 14% to 33% of the general population, and 17% to 33% of the Asian population, giving rise to economic burdens to both the patient’s family and society [2,3]. According to the structure and function of the tear film, dry eye is clinically divided into two major types: reduced tear production and the increased evaporation of the tear film [4]. These conditions are not mutually exclusive; in fact, they often overlap. Patients with reduced tear production usually show a low Schirmer I test result, and patients with increased evaporation of the tear film show a tear film break-up time (TFBUT) that is reduced. Ocular surface damage and elevated tear film osmolarity can occur in both forms [5]. Recent studies found that the pathophysiology of DED is complex, and dry eye is a multifactorial and self-perpetuating inflammatory disease [6]. Both intrinsic factors, such as female gender, aging, Asian ethnicity, Sjögren’s syndrome, and systemic diseases, and extrinsic factors, such as the extensive use of cell phones and computers, environmental pollution, smoking, drug use, local inflammation of the eye, eye surgery, and contact lens wearing, can contribute to instable tear film [7] and increased tear osmolarity. Consequently, the stress signaling pathways are activated and inflammation is initiated [8].

It is necessary to distinguish dry eyes from eye infections and allergies through accurate diagnosis. If epitheliotoxic antibodies or antiallergic drugs are prescribed on the basis of an incorrect diagnosis, the symptoms of dry eyes may worsen [5]. The clinically available tests are summarized in Table 1. Tear osmolarity, which is superior to almost all other DED tests, is considered a reliable diagnostic test for detecting dry eye [9,10]. Tear secretion and the tear film break-up time, corneal staining score, conjunctival staining score, tear osmolarity, and questionnaire score are other clinically available methods for diagnosing dry eye [11,12]. Although these tests are routine and necessary for diagnostics in the detection of dry eye disease, it is difficult to obtain high specificity and high sensitivity simultaneously. Moreover, due to the multifactorial pathogenesis of dry eye, it is difficult to standardize the diagnosis of dry eye. The determination of the physiological characteristics and biomarkers of tears, including the pH (H_3_O^+^/OH^−^) [13], electrolytes (Na^+^, Cl^−^) [13], lysozyme [14], lactoferrin [14,15], interleukin proteins (IL-1α, IL-1β, and IL-17) [16], tumor necrotic factor (TNFα) [17], interferon gamma (IFNγ) [18], matrix metalloproteinase-9 (MMP-9) [19], and mucins [20], can provide a more critical determination of DED. These tests, however, are limited by the need for specialized equipment, poor environmental stability of protein assay kits, or the use of expensive molecular detection reagents (i.e., antibodies). Therefore, there is an urgent demand for faster, more affordable, and easy-to-use biosensors that can be used to diagnose dry eyes.

Based on the symptoms and the clinical exam findings, DED is classified into mild, moderate, and severe levels. The treatment of dry eyes depends on the pathophysiology and severity level. Clinically, drugs for the treatment of DED include (I) lubricating agents, such as artificial tears and sodium hyaluronate eye drops; (II) anti-inflammatory drugs, such as glucocorticoids, cyclosporine A, and non-steroidal anti-inflammatory drugs; (III) sex hormones; (IV) secretagogues, such as pilocarpine eye drops; and (V) autologous serum eye drops [5,21,22]. Given its noninvasiveness and ease of administration, the topical administration of eye drops is the most common early treatment method for dry eyes. However, due to the complex structure of the eye surface, rapid clearance caused by tear dilution and the lacrimal drainage system leads to a low bioavailability, short retention time, and, subsequently, low efficacy [23]. An increased dose is often adopted to address this problem, which unfortunately increases the risk of systemic side effects due to the conjunctival absorption of drug molecules by the blood through the nasolacrimal system [24]. Severe dry eyes can be treated by surgery, such as autologous gland duct transplantation, palpebral suture, amniotic membrane transplantation, and submandibular and labial gland transplantation [25,26]. Therefore, the early cure of dry eyes through control-released drugs is promising.

Hydrogels are highly hydrophilic materials with a three-dimensional polymer network, which swell rapidly in water and maintain a large volume of water without dissolving this swelling state [27]. A wide range of natural, semisynthetic, and synthetic polymers can be used as starting materials for hydrogels [28]. Chitosan, hyaluronic acid (HA), carbopol, sodium alginic acid, poly(ethylene glycol) (PEG), poly(vinyl alcohol) (PVA), acrylic polymers, and siloxanes are representative polymers for synthetic gel-forming materials [29,30,31,32,33,34]. Drug-loading platforms, such as nanoliposomes and solid nanoparticles, can be incorporated into hydrogels for disease treatment [35,36]. The methods used for preparing hydrogels include chemical cross-linking (covalent bond) and physical cross-linking (non-covalent bond) [37]. Chemical cross-linking can lead to good mechanical strength in the hydrogels [38]. It is worth noting that, some small-molecule chemical crosslinkers and initiators are not environmentally friendly or are even toxic [39]. Physical cross-linking is usually achieved by means of electrostatic interaction, hydrophobic interaction, hydrogen bonding, etc., which can be reversible and rapidly rebuilt without any external factors [40].

The designed hydrogels can have good biocompatibility, excellent physical and mechanical properties, and long-term implant stability. Much emphasis has been placed on research on hydrogel as an ophthalmic biomaterial, and its main applications include long-term drug delivery on the ocular surface [41], the sustained release of long-acting drugs in the eye [42], corneal injury repair [43], artificial vitreous [44], and so on. These hydrogels can be retained in the eyes for a long time, reducing the adverse reactions caused by the systemic absorption of the drug, and their tolerance is better than that of ointment. Importantly, hydrogels have been studied in drug delivery because they can hold many different substances in the crosslinked matrix. They range from hydrophobic and hydrophilic molecules to micromolecules and macromolecules. Polymer microgels and nanogels, as non-imprinted hydrogels capable of semi-selectively recognizing proteins, do not match antibodies in terms of selectivity. Still, they are highly adjustable, based on inexpensive materials, and environmentally robust, suggesting that they are favorable substances for treating ocular disease. Moreover, we can implant these biosensors into hydrogel contact lenses (CLs) to continuously monitor the tear status [45].

Hydrogels are emerging drug-delivery platforms which offer many advantages for diagnosing and treating DED. The good transparency and biocompatibility of hydrogels are prerequisites for their applications in the field of ophthalmology [46]. Hydrogels have great prospects for application in the improvement of corneal permeability, drug bioavailability, and the prolongation of the drug retention time on the ocular surface [47]. Moreover, hydrogels can be used as materials for contact lenses which can not only provide protection and hydration but also be designed as drug carriers to improve the bioavailability and prolong the retention time of drugs on the ocular surface. In addition to the protective and hydrating functions of hydrogel contact lenses, the drug-loaded hydrogel contact lenses can prolong the retention time of the drug on the ocular surface [48,49]. There is a highly controversial issue between dry eye treatment with CLs and dry eye patients. It is widely thought that dry eye is more common among CLs wearers. Interestingly, dry eye can be relieved and treated by adding some drugs to CLs, including rewetting agents or anti-inflammatory drugs [50,51].

The past decade has witnessed significant headway achieved in research on the etiology of dry eye, which has led to the emergence of many clinical therapeutic drugs, thus significantly improving the quality of life of patients with dry eye. The hydrogels used in ophthalmology include artificial tears, drug carriers, CLs, adhesives, etc. Here, we review the recent advances in the diagnosis and clinical treatment of dry eye with hydrogels.

## 2. Application of Hydrogels in the Diagnosis of Dry Eye

Interestingly, fluorophores or biosensors can be combined with hydrogel contact lenses to detect electrolytes in the tears (Table 2). Such so-called smart contact lenses (SCLs) can provide information on tear biomarkers or tear evaporation for a certain period of time. The hydrogel-based SCLs provide a solution for the clinically detection of DED biomarkers and the diagnosis of DED. Despite the fact that SCLs can continuously detect and monitor multiple tear biomarkers of dry eyes, it is challenging to integrate batteries for long-term power supply due to the limited area of SCLs. Other aspects, such as the specificity, sensitivity, integration of readout circuits, and repeatability, also need to be improved. Once these issues are resolved, biosensors based on a hydrogel system are expected to be beneficial for the personalized medical treatment of DED.

### 2.1. High-Isoelectric-Point Proteins

**Figure 1:**
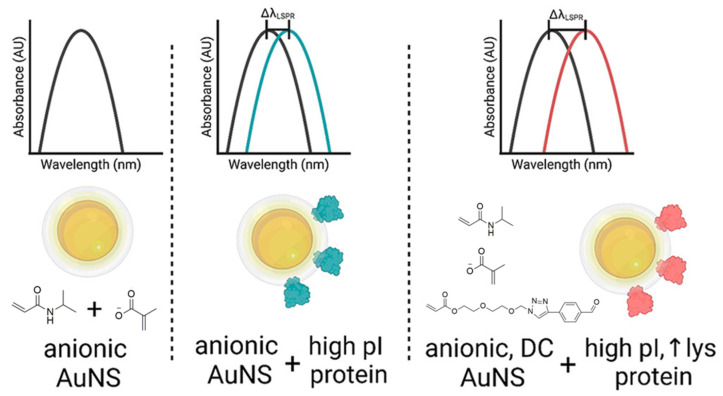
Schematic depiction of the binding of AuNS to high-isoelectric-point proteins. Reprinted with permission from Wechsler et al. [14], *Nano Lett.*, published by ACS Publications, 2021.

Lysozyme, lactoferrin, IgA, and Lipocalin-1 make up 70–85% of the proteins in tears [52]. Lactoferrin is a multifunction chain polypeptide with properties such as anti-inflammatory, bacteriostatic, and antioxidant effects [53]. Lysozyme is a glycolytic enzyme with antimicrobial functions [54]. The lysozyme and lactoferrin levels are significantly decreased in dry eye patients. Tear film lysozyme and lactoferrin can be considered as simple, non-invasive, and economical biomarkers for diagnosing dry eye [55,56]. Among the four proteins, lysozyme and lactoferrin are high-isoelectric-point proteins, while the isoelectric points of IgA and Lipocalin-1 are lower than the neutral pH value. Poly(nisopropylacrylamide-co-methacrylic acid) (P(NIPAM-co-MAA))-based hydrogels coated gold nanoshell (AuNSs) were used to develop a label-free biosensor for detecting high-isoelectric-point tear biomarkers associated with dry eye. A custom, aldehyde-functionalized oligo(ethylene glycol)acrylate (Al-OEGA) was included in the hydrogel coating to enhance protein recognition by forming a dynamic covalent (DC) imine bond with the lysine residues on the surfaces of higher-electric-point proteins, thus improving the sensitivity of the biosensor. The binding of AuNS to higher-isoelectric-point proteins in hydrogels resulted in a concentration-dependent change in the value of localized surface plasmon resonance Δ λLSPR (Figure 1). Importantly, this contributes to the development of low-cost biosensors for dry eye and has the potential to become an affordable method of dry eye detection [14].

A local-surface-plasmon-resonance (LSPR)-based biosensor for protein detection was developed using silica gold nanoshells (AuNSs) (AuNS@PNM) coated with ionic poly(nisopropylacrylamide-co-methacrylic acid) (PNM) [57]. Intriguingly, AuNS@PNM could detect changes in the concentrations of two protein biomarkers in chronic dry eye: lysozyme and lactoferrin. Both proteins reportedly have high isoelectric points, resulting in electrostatic attraction between the negatively charged PNM hydrogels and positively charged proteins. AuNS@PNM exhibited an LSPR red-shift of a magnitude of up to 50 nm that was concentration-dependent upon lysozyme or lactoferrin binding. Notably, the materials and signal transduction methods used in this biosensor were low-cost. It required only a few microliters of tears, and the test could be performed in a few minutes with a regular tablet reader. Accordingly, it was possible to detect changes in the concentrations of these two biomarkers in human tears.

### 2.2. Electrolytes

Tear osmotic pressure is considered to be a reliable indicator of dry eye. Electrolytes, including Na^+^, K^+^, Ca^2+^, pH (H_3_O^+^/OH^−^), Cl^−^, and so on, are considered to be the main components maintaining the osmotic pressure of tears. Each type of tear has different concentrations of electrolytes. Changes in the concentration of tear electrolytes have been proven to be correlated with dry eye disease [58]. Therefore, the detection of the electrolyte concentration in tears is of great value for the diagnosis of DED. Several methods have hitherto been designed to detect electrolytes in tears. On the one hand, there is too little basic tear on the eye surface (ca. 6 µL/eye); on the other hand, contact with the eye surface can stimulate the secretion of tears and change the tear volume, leading to inaccurate test results [59]. It has been reported that the ion concentration in tears is calculated by instantly measuring the electrical conductivity of tears [60]. However, this method can only provide a momentary tear detection result and cannot provide the concentration of a single ion. Moreover, the detection method used to detect tear proteins is unsuitable for the detection of the tear electrolyte concentration. Interestingly, the Badugu team explored the development of hydrogel contact lenses. Fluorophore, combined with hydrogel contact lenses, was used to detect the concentrations of ions in tears.

The Badugu team initially created pH- (H_3_O^+^/OH^−^) and Cl^−^-sensitive SiHG contact lenses. They linked the hydrophobic C18 chain to a water-soluble fluorescent probe to obtain hydrophobic ion-sensitive fluorophores (H-ISF) so as to determine the pH and chloride. The H-ISF and SiHG lens combination was potent and could not be washed off by an aqueous solution. The measurements of the pH (H_3_O^+^/OH^−^) and Cl^−^ did not affect each other. An adequately designed H-ISF could allow hydrogel contact lenses to measure the tear electrolyte levels [13]. Subsequently, the Badugu team studied Na^+^-sensitive fluorescent hydrogel contact lenses. Furthermore, Na^+^- and Cl^−^-sensitive fluorescent groups combined in different hydrogel contact lenses were studied. The Na^+^- and Cl^−^-sensitive fluorophores were derived and combined in a non-covalent manner with two commercial silicone hydrogel (SiHG) contact lenses: Biofinity (ComfilconA) or MyDay (StenfilconA) contact lenses. The combination of Na^+^ and Cl^−^ with the CLs was completely reversible. The levels of Cl^−^ sensitivity of the two lenses were similar, but the range of the Na^+^ sensitivity was different between Biofinity and MyDay lenses. Moreover, lenses loaded with both Na^+^ and Cl^−^ probes in a single MyDay lens were designed, which could independently measure the Na^+^ (490 nm) and Cl^−^ concentrations (454 nm) at different wavelengths [61]. The Badugu team further developed Na^+^-sensitive fluorescent hydrogel contact lenses. They combined three sodium-sensitive fluorescent groups (SG-C16, SG-LPE, and SG-PL) derived from 1-hexadecyl amine, 1-oleoyl-2-hydroxy-sn- glycerol-3-phosphate ethanolamine, and poly 1-lysine in SiHG contact lenses. The intensity of the probe changed with the Na^+^ concentration, which was used to detect the concentration of sodium ions in the tears [62]. The successful development of fluorescent hydrogels that are sensitive to pH (H_3_O^+^/OH^−^), Na^+^, and Cl^−^ provides a roadmap for the development of hydrogel contact lenses that are sensitive to other related electrolytes in tears.

### 2.3. Tear Evaporation

It has been established that commonly used tear secretion detection methods, such as Schirmer and Phenol Red thread tests, cannot reflect the continuous detection results in different environments of the eye, increasing the risk of the misdiagnosis of DED. As the rapid evaporation of the tear film causes DED, a tear evaporation test can also diagnose DED. In the case of smart contact lenses (SCLs), biosensors were loaded into hydrogel contact lenses to continuously monitor certain parameters while avoiding any irritation or toxicity resulting from the long-term operation. SCLs have shown significant advantages in recent years, as they can noninvasively and continuously sample the tear fluid, analyze various dry eye biomarkers, and transmit data for remote analysis [63]. Chiou et al. [45] designed a SCL sensor system to continuously evaluate tear evaporation. The SCL system consisted of an adjustable sensitivity sensor readout circuit, a tear sensor, and an antenna and was embedded in biocompatible hydrogel contact lenses through a commercial manufacturing process. The subjects were instructed to wear the SCL for continuous tear monitoring. Since the SCL system with a radio frequency identification (RFID) reader interface could record the tear volume based on changes in the capacitance or resistance rather than the tear weight, and a 5.3% change in the capacitance accounts for <0.01% tear weight loss, it was considered an ultra-sensitive high-resolution method for the continuous measurement of tear evaporation.

## 3. Hydrogel Eye Drops for Dry Eye Treatment

The topical use of artificial tears is the first-line treatment for dry eye. Artificial tears can play a similar role to human tears, lubricating the ocular surface, increasing its wettability, diluting the soluble inflammatory mediators, and reducing the tear’s osmotic pressure, thereby exerting a therapeutic effect [64]. However, artificial tears can quickly be removed from the ocular surface (in less than 5 min) due to reflex tearing, blinking, and rapid anterior corneal clearance, giving rise to low bioavailability [20,65]. The frequent instillation and increased viscosity of artificial tears can solve this problem to a certain extent but can also lead to poor patient compliance [66]. Unlike these conventional eye drops, the ophthalmic hydrogel, a shear-thinning non-Newtonian fluid with an elastic modulus greater than the viscous modulus, has a certain yield stress and thixotropy. That is, hydrogel eye drops show high viscosity under low shear, which is beneficial for their stability and uniformity during storage. Under high shear (i.e., when applied), the viscosity decreases rapidly, which is conducive to the application of the hydrogel to the eyes and to improving wearer comfort [67]. The recent patents of hydrogel products for the treatment of DED are shown in Table 3. Table 4 summarizes a number of artificial tears, including Hylo^®^ Gel (URSAPHARM, S aarbrück-en, Germany), Vidisic^®^ gel (Bausch & Lomb, Rochester, NY, USA), GelTears^®^ (Bausch & Lomb, Rochester, NY, USA), Viscotears^®^ (Novartis, Basel, Switzerland), and Clinitas Gel^®^ (Altacor, Reading, UK), which are already on the market or are at different clinical developmental stages.

### 3.1. Ocular Lubricating Polymers

**Figure 2:**
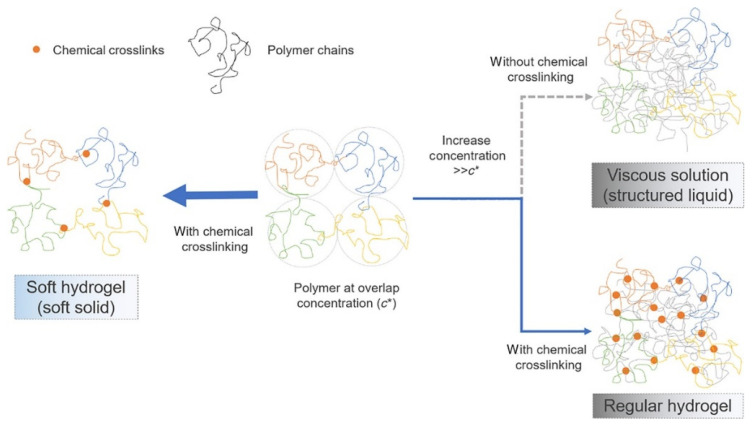
Illustration of the difference between a soft hydrogel, a viscous solution, and a conventional hydrogel. The orange dots represent crosslinks, and the threads of different colors represent different polymer chains. Center: a polymer solution where the polymer concentration is at the level of the overlap concentration (c*), where the polymer chains are just dense enough to touch each other. The dotted circles represent the average space (blob size) that one polymer occupies. On the right: with the polymer concentration increasing above c*, the polymer chains become increasingly entangled, and the solution turns viscous. A total of 16 conventional hydrogels are formed by crosslinking the polymers under such conditions. On the left: the chains of a polymer solution at a concentration of approximately c* are crosslinked to form a hydrogel of the lowest possible crosslinking density for such a polymer. Reprinted with permission from Yu et al. [76], *Bioengineering & Translational Medicine*, published by Wiley (Hoboken, NJ, USA), 2021.

A variety of ocular lubricating polymers, such as sodium hyaluronan (hyaluronic acid, HA), methylcellulose, poly(vinyl alcohol) (PVA), polyvinylpyrrolidone (PVP), poly(acrylic acid) (PAAc), have been used in artificial tears [70]. As a natural polysaccharide found in vitreous and aqueous humor, tears, lacrimal glands, conjunctiva, and corneal epithelium [71], HA has attracted considerable attraction due to its good biocompatibility and biodegradability. HA-based materials can be used as effective drug carriers for dry eye disease treatment due to their excellent biocompatibility, biodegradability, bioadhesive properties, viscoelasticity, and receptor interaction characteristics [26]. Moreover, HA solution is a non-Newtonian fluid, exhibiting shear-thinning and viscoelastic behavior [72]. It has been established that crosslinking HA covalently can prolong the retention time of HA in the cornea [73]. Williams et al. [74,75] developed a crosslinked hydrogel based on thiolated HA (xCMHA-S), which exhibited a stable shear viscosity, and this hydrogel eye drop could reportedly prolong the tear film break-up time in rabbits, enhance tear film stability, and significantly reduce dry eye in dogs for two weeks when applied twice daily. Moreover, xCMHA-S effectively reduces the dosing frequency and is more effective than conventional replacement tear supplements. In another study by Yu et al. [76], under a theoretical framework derived from the Blob model, high-molecular-weight HA molecules (120 kDa to 2.6 MDa) modified with the respective vinylsulfone (VS) and thiol (SH) groups were crosslinked to create synthetic soft hydrogels. The rheological properties of the prepared hydrogels were dominated by elasticity under low shear stress and by viscosity when the stress exceeded a small threshold (Figure 2). The hydrogel remained on the ocular surface for at least 5 h. Applying the soft hydrogel in combination with CsA twice a day for one month improved the clinical symptoms of more than 65% of the canine patients who had not responded to previous CsA monotherapy.

**Figure 3:**
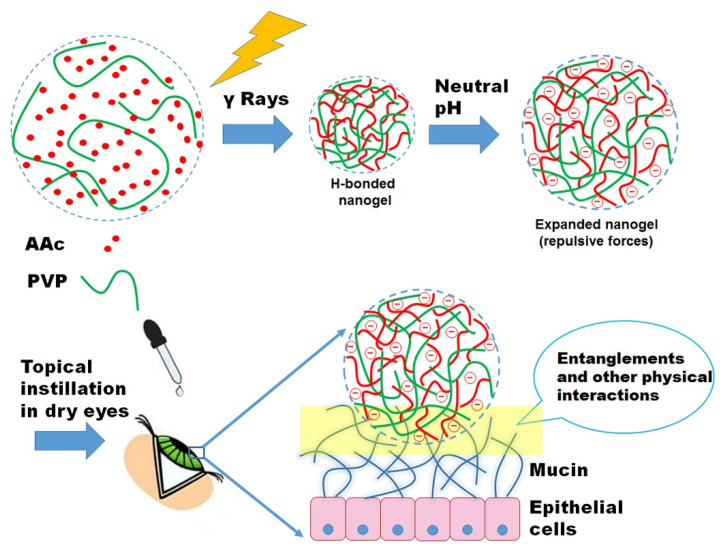
Proposed mechanism for the preparation of PVP/PAAc nanogel and its interaction with mucin. Reprinted with permission from Swilem et al. [77], *Mater. Sci. Eng. C Mater. Biol. Appl.*, published by Elsevier (Amsterdam, The Netherlands), 2020.

When topically applied to the eye, PAAc, in either the linear or 3D cross-linked form, instantly forms a lubricating film over the conjunctiva and cornea, which persists for a long time. However, this polymer usually forms highly viscous gels at the usual concentrations (about 0.2%) under physiological conditions similar to those of the ocular surface, which results in the transient blurring of the vision, a sticky sensation, and reduced patient compliance. Swilem et al. [77] prepared nanogels composed of PAAc and PVP by adapting an ionizing radiation method. The nanogels’ formation was driven by the hydrogen-bonding complexation between PVP and PAAc (formed in situ), as well as the radiation-induced cross-linking. The dry eye model showed that the twice daily instillation of PAAc-rich nanogels prepared at 20 kGy improves eye dryness more efficiently and rapidly (3 days) (Figure 3).

### 3.2. Temperature-Sensitive Hydrogels

Various environmentally sensitive hydrogels, such as temperature-sensitive, pH-sensitive, and ion-sensitive ophthalmic gels, have been developed for ocular administration to improve the bioavailability [78,79]. The temperature-sensitive hydrogel can undergo reversible sol–gel transition following temperature changes. When the temperature is lowered, it is in a sol state (similar to a liquid) and changes into a gel state at higher temperatures [80]. Commonly used temperature-sensitive polymers include poly(N-isopropyl acrylamide) (PNIPAAm) and polyethylene glycol (PEG). The thermally reversible phase transition temperature of PNIPAAm is 32 °C, close to the human body surface temperature [81].

**Figure 4:**
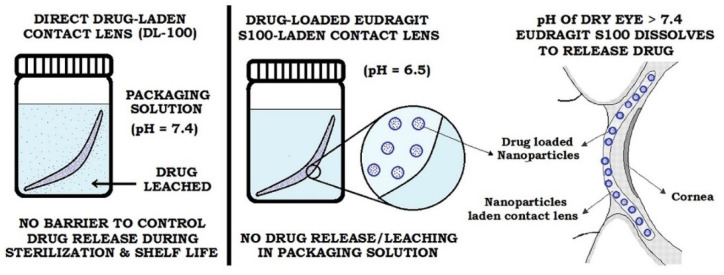
pH-triggered drug delivery through contact lenses. Reprinted with permission from Maulvi et al. [104], *Colloid Surface B*, published by Elsevier, 2017.

A number of PNIPAAm-based drug delivery hydrogels have been developed to treat DED. The encapsulated drugs include epigallocatechin gallate (EGCG), CsA, Tacrolimus (also known as FK506), and pimecrolimus (PMS). Luo et al. [82] designed a sustained-release and mucoadhesive drug delivery system that is effective for local DED treatment. Specifically, the temperature-responsive hydrogel was composed of gelatin, PNIPAAm, and lectin *Helix pomatia* agglutinin, which enabled the sustained release of the encapsulated EGCG molecules. The one-time instillation of an EGCG-loaded carrier onto the conjunctival sac of rabbits could sustainably and effectively repair the corneal epithelial defects and reduce cellular inflammation, oxidative stress, and apoptosis for over 14 days. Wu et al. [83] synthesized HA-grafted PNIPAAm, a thermosensitive copolymer, which could be used as a potential carrier for the topical application of CsA. The HA-g-PNIPAAm microgels exhibited a high drug loading efficiency (73.92%) and controlled release properties. FK506, a macrolide antibiotic, is a potent new immunosuppressant that relieves xerophthalmia by inhibiting the release of interleukin-2 (IL-2) and comprehensively inhibiting the role of the T lymphocytes [84]. However, the strong hydrophobicity of FK506 can adversely affect the efficacy. Han et al. [85] developed a copolymer composed of PEG, polypropylene glycol (PPG), and polyhedral oligomeric silsesquioxane (POSS), called MPOSS-PEG-PPG (MPEP). The MPEP hydrogels could enwrap FK506 in the hydrophobic core of the formed micelles to improve the water solubility, thereby providing a viscous, long-acting delivery system for FK506. It has been shown that the MPEP-FK506 hydrogel can effectively relieve dry eyes in mice (Figure 4). PMS is an ascomycin macrolactam derivative that inhibits the transcription and release of proinflammatory cytokines in T cells. Fan et al. [86] designed a thermosensitive nano-hydrogel containing PMS, which displayed special gel–sol transition behavior as the temperature increased from 4 °C to 37 °C and enabled the slow release of PMS to improve the dry eye symptoms in mice.

In addition to PNIPAAm, more thermosensitive materials have been fabricated. Lin et al. [87] developed a thermosensitive chitosan-based hydrogel as a new intracapsular plug for intracapsular injection in the treatment of dry eye. This thermosensitive hydrogel system was based on a new derivative of chitosan, hydroxybutyl chitosan (HBC), which forms a mild viscous solution at low temperatures. At temperatures above 20 °C, the system quickly transformed into a flexible and durable hydrogel. The thermosensitive HBC hydrogel can be used as a new absorbable intraocular embolic agent to treat dry eye through a new “liquid plug” strategy.

## 4. Hydrogel Contact Lenses for Dry Eye Treatment

Over the years, hydrogel contact lenses have been widely used in ophthalmology. A summary of recent advances of drug-loaded hydrogels in the treatment of dry eye disease is listed in Table 5.

It is well-established that hydrogel contact lenses have a good biocompatibility, physical and mechanical properties, good water vapor, and suitable gas (such as O_2_ and CO_2_) transmittance [112,113]. Moreover, hydrogel contact lenses are very convenient in terms of their application and removal from the eyes, as they do not adhere to the wound and can sense minute changes in external stimuli [114,115,116]. Moreover, the excellent porosity for drug loading highlights the great potential of the hydrogel contact lens as a drug delivery system. Compared with conventional eye drops, CLs enable sustained drug release and do not affect the patient’s vision [117]. The tear film formed between the CLs and the cornea can effectively enwrap the drug, prolong the retention time, and prevent the drug from being diluted by tears, thereby improving the drug delivery efficiency [118]. In addition, the CLs delivery system can continuously release low doses of drugs, avoiding the side effects of high-dose administration. The drugs used in hydrogel contact lenses to treat dry eye generally include tear substitutes or wetting/lubricants, anti-inflammatory drugs, osmotic protective agents, tear secretory agents, and so on [119].

Different approaches have been developed in order to load drugs into contact lenses to treat DED. Soaking is the simplest and most cost-effective method. The drug is loaded by soaking the contact lenses in a drug solution and released through the front and back of the lens in the tear film [120]. High-molecular-weight drugs cannot penetrate the water channel of contact lenses [121,122]. Multiple therapeutics can also be loaded into the hydrogel contact lenses using the molecular imprinting technique, wherein functional monomers are mixed with therapeutics before polymerization, achieving a high drug affinity and increased drug loading capacity [123]. Moreover, the drug release mode can be customized according to the monomer composition. The incorporation of drug-loaded colloidal nanoparticles into contact lenses may also regulate drug release over a long period of time [124]. However, decreased ion and oxygen permeability and an increased storage modulus may also be observed in hydrogel contact lenses [125].

As a result of the introduction of hydrogels into medical therapy, the factors involved in the process scale-up, such as the technical considerations, medical device regulations, and economic feasibility study, must be considered [126]. The ophthalmic application of hydrogels in the form of drug-eluting contact lenses is still incipient at the clinical level. Although the use of soft contact lenses for drug delivery has been extensively studied for more than 20 years, only ACUVUE^®^ Theravision™ (Johnson & Johnson Vision Care, Inc., Jacksonville, Florida, USA) with Ketotifen, the world’s first and only drug-eluting contact lens, has been recently approved by the FDA [127]. The approval is encouraging for the development of drug-eluting hydrogel contact lenses that could be used to treat eye diseases. In the United States (US), mass-produced contact lenses are regulated by the Food and Drug Administration (FDA). Daily wear soft lenses and rigid corneal lenses are considered as Class II medical devices (moderate- to high-risk), while overnight and myopia management contact lenses are considered as Class III medical devices [128]. The regulations of CLs in the European Union, Australia, India, and China are similar to those in the US. With the rapid development of polymer materials, the safety of new materials should be systematically evaluated. Tissue toxicity may also arise after the long-term application of non-degradable polymers to the ocular surface. 

### 4.1. Lubricating and Rewetting Agents

A common method used to relieve dry eye discomfort is the application of lubricating and rewetting agents to lubricate the eye surface and increase its humidity. Importantly, the sustained release of lubricating and rewetting agents from CLs can prolong the retention time of the encapsulated drug on the eye surface.

HA molecules play an important role in maintaining the functions of eyes. HA and rod-like collagen create a fiber-reinforced composite in the vitreous, maintaining the shape and mechanical stability of the eyes and enabling the selective diffusion of nutrients and solutes [129]. Additionally, they are natural boundary lubricants in vivo. The covalent addition of HA to hydrogel contact lenses can improve the wettability, reduce protein deposition, and reduce friction [95,96]. HA is a common anti-xerophthalmic drug used in ophthalmology. It can accelerate the repair of corneal epithelial cells and increase eye moisture [76]. HA, as an internal wetting agent, when coated on hydrogel contact lenses, can reduce lysozyme adsorption and denaturation [130,131]. Interestingly, Yamasaki et al. [94] improved the hydrophilicity of CLs by combining a low-molecular-weight HA derivative containing hydrophobic groups and soft contact lenses.

Maulvi et al. [100] loaded HA into hydrogel contact lenses by impregnation, and they released HA within 10 days of treatment. HA-laden hydrogel contact lenses prepared by the direct encapsulation method showed significant sustained release [98]. HA was modified with siloxy using click chemistry to improve the CL surface properties. Siloxane-modified HA significantly reduced lysozyme adsorption to 20% and 16% in traditional and silicone hydrogel contact lenses [132,133]. Thiolated HA reduced the contact angle, dehydration rate, and non-specific adsorption of lysozyme and albumin to alleviate dry eye and contact lens discomfort [97]. Akbari et al. [99] loaded HA onto chitosan nanoparticles (NPs) and then dispersed the HA-loaded NPs in a cyclic PVA hydrogel to prepare a new type of ring implant PVA contact lenses, which showed a desirable rheological stability under physiological shear force. The release of HA from the ring implant CL lasted for 14 days. Maulvi et al. [134] designed a novel corneal ring implant contact lens containing HA by an improved casting method to alleviate benzalkonium-chloride-induced dry eye in rabbits. Ali et al. [135] designed and synthesized hydrogel contact lenses using molecular imprinting technology, and it was determined that the lenses can release HA at a controllable rate of 6μg/h for 24 h.

Hydroxypropyl methylcellulose (HPMC) is also a wetting agent used in CLs. White et al. [136] loaded 120 kDa HPMC onto silica hydrogel lenses by the molecular imprinting technique and released approximately 1000 µg HPMC at a constant flow rate of 16 µg/day for 60 days during the whole continuous wearing period. Kim et al. [101,102] loaded HPMC into the pH-sensitive hydrogel contact lens, which can regulate the release of HPMC through changes in the pH and is effective against dry eye disease.

Current evidence suggests that mucins serve as excellent boundary lubricants, and the loss of mucin on the eye surface can cause damage to the surface epithelial cells [137]. Contact lenses coated with purified gastrin mucins can effectively prevent corneal damage, improve the performance of the hydrogel lens, provide an automatic lubrication mechanism when in contact with the eyes, and effectively protect the eye against dry eye [111].

Electro-osmotic flow (EOF) has been reported as a new anti-dehydration mechanism in CL, which can be driven by built-in biocompatible power sources, such as enzyme batteries. The CL formed from charged hydrogel is used as the liquid pipeline produced by EOF. Upward EOF within the CL can effectively maintain the moisture of the lens and overcome the negative effects of dry eye syndrome [138].

### 4.2. Anti-Inflammatory Drugs

Inflammation has been observed at all stages of DED. Baudouin et al. [139] put forward the concept of a “vicious circle of inflammation” and believed that inflammation plays a vital role in the pathogenesis of dry eye. Several anti-inflammatory treatments have been used to treat dry eye, including cyclosporine A (CsA), corticosteroids, and non-steroidal anti-inflammatory drugs. These drugs inhibit the expression of inflammatory mediators on the ocular surface, restoring the secretion of healthy tear films and reducing the signs and symptoms.

CsA is an immunosuppressant with potent effects and high selectivity, which was used to treat DED in the 1990s. However, CsA may cause side effects such as eye pain and hyperemia, with a poor tolerance, low bioavailability, and drug instability. Therefore, great efforts must be made to reduce the side effects of CsA [140]. Peng et al. [141] loaded CsA and vitamin E into silicone hydrogel (SiH) contact lenses by soaking. SiH contact lenses can release CsA for about 1 month. Choi et al. [103] used nanoporous silica as a drug carrier and mixed nanoporous silica with CsA to prepare CsA-eluting CLs using supercritical fluid technology, and the CLs could release CsA continuously for 48 h, exhibiting good eye penetration, improving the clinical parameters and conjunctival goblet cell density, and reducing the number of inflammatory cytokines. Maulvi et al. [104] designed novel cyclosporine-loaded Eudragit S100 (pH-sensitive) nanoparticle-laden contact lenses by the free radical polymerization method to continuously release CsA at a therapeutic rate. In vivo studies of rabbits showed that the sustained release of tears could last for up to 14 days (Figure 4).

Silicon-based hydrogel contact lenses soaked in vitamin E can enhance the loading capacity of betamethasone (BMZ), a commonly used and effective drug for the treatment of ocular inflammation, and significantly extend the release period of 90% drug molecules to 600–624 h [109]. White et al. [110] designed SiHG contact lenses through a molecular imprinting strategy, which can release up to four template molecules simultaneously, including HPMC, trehalose, ibuprofen, and prednisolone. The release rate can be effectively controlled by adjusting the ratio of functional monomers to comfort molecules, which can address the multiple propagators of contact lens discomfort.

**Figure 5:**
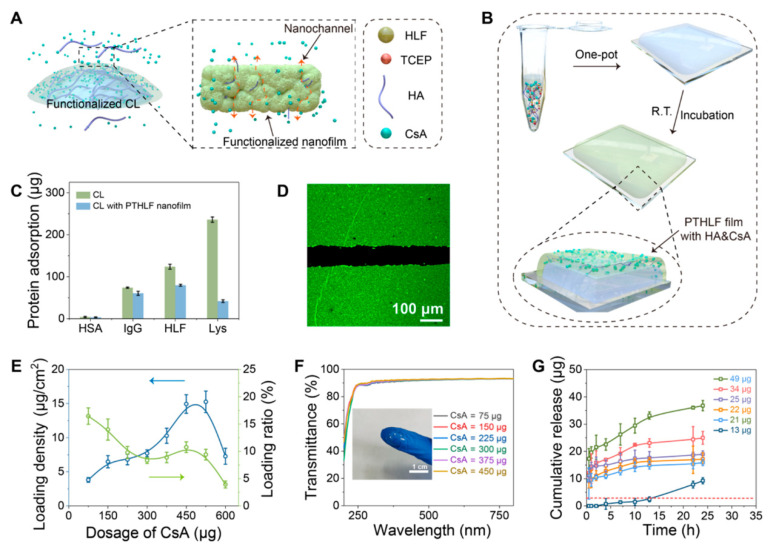
Encapsulation and release of CsA and HA in the PTHLF film. (**A**) Schematic cartoon showing the release process of functional molecules from the functionalized CL with the PTHLF-coated nanofilm. (**B**) Schematic illustration showing the one-pot encapsulation of CsA and HA in the nanofilm prepared by simply mixing HLF (7 mg/mL in Milli-Q water (Millipore Corporation, Billerica, MA, USA)), TCEP (50 mM in Milli-Q water), CsA (7.5 mg/mL in aqueous ethanol), and a solution of HA and incubating the solution for 12 h at room temperature. (**C**) Evaluation of the nonspecific adsorption of the proteins on the bare and PTHLF-coated CL surfaces (tested using a bicinchoninic acid (BCA) assay). (**D**) LSCM image of the PTHLF film encapsulating CsA-FITC. (**E**) Experimental loading density (blue line) and loading ratio (green line) of CsA in the PTHLF film at different feeding doses of CsA. (**F**) Optical transparency of the functionalized PTHLF film coated on quartz glass (inset: a photograph of the functionalized CL). (**G**) Release curve of the encapsulated CsA from the functionalized film. Reprinted with permission from Qin et al. [105], *ACS Cent. Sci.*, published by ACS, 2022.

Qin et al. [105] reported a transparent and biocompatible amyloid-like nanofilm that can destroy the hydration layer of a wet surface and achieves strong adhesion to a hydrogel/tissue surface within 2 s. Based on this conceptual progress, a functional therapeutic CL was fabricated by adhesion to a functionalized phase-transitioned human lactoferrin (PTHLF) nanofilm (carrying CsA) on the CL surface for DED treatment. The PTHLF nanofilms were adhered to the hydrogel CL surface by manipulating the amyloid-like aggregation. These therapeutic CLs displayed excellent therapeutic efficacy, whereas a ≥82% increase in the CsA bioavailability was obtained when compared to the commercial pharmacologic treatment (Figure 5).

### 4.3. Secretagogue

Diquafosol is a commercial secretion agent for the treatment of dry eye. Its formulation is based on 3 mM uridine tetraphosphate (Up4U), a dinucleotide that acts on the P2Y2 receptors on the conjunctival epithelial cells and goblet cell membranes, promoting the secretion of conjunctival epithelial cells and goblet cell mucin, and increases the stability of the tear film. Dominguez-Godinez et al. [106] soaked two commercial silicone hydrogel (Si-Hy) CLs (comfilcon A and balafilcon A) in a diquafosol solution overnight for 12 h to cause the two kinds of CLs to release diquafosol continuously for 5 h, so as to increase the retention time on the ocular surface and promote tear secretion for a long time. Another dinucleotide, diadenosine tetraphosphate (Ap4A), was also loaded into hydrogel contact lenses by immersion. The release of Ap4A from the non-ionic silicone hydrogel contact lenses lasted for 300 min [107].

### 4.4. Osmoprotectant

High tear film permeability is the primary symptom of dry eye. Osmoprotectants, such as L-carnitine and betaine, can play an electrolyte-like role in balancing osmotic pressure and helping cells to survive in high osmotic conditions [142]. Hsu et al. [108] developed a silicone hydrogel contact lens loaded with 20–23% vitamin E, which could control the release of betaine to reduce the symptoms of eye dryness. Betaine is a small molecular compound that diffuses easily in CLs and is difficult to retain for a long time. The diffusivity of betaine decreases slightly upon the incorporation of vitamin E into the lenses because a small proportion of the vitamin E loaded into the gel is dissolved in the lens matrix and renders the lens more hydrophobic, while the remaining vitamin E forms diffusion barriers. Importantly, loading with 20% vitamin E can extend the betaine release time to about 10 h, which is 60 times longer than that of control contact lenses.

### 4.5. Bandaged Contact Lenses

Li et al. [143] demonstrated the efficacy of Balafilcon silica gel contact lenses as bandaged contact lenses (BCL) in the treatment of severe dry eye caused by Sjögren’s syndrome (SS). Even when the eye surface is poor, this bandage contact lens is safe and effective. BCLs have been shown to decrease the necrosis and desquamation of the corneal epithelium by preventing blink-associated mechanical stress and promoting the subsequent acceleration of wound healing. In addition to moderate corneal epithelium function, this treatment can significantly relieve dry eye symptoms. These long-worn SCLs may be used to treat dry eyes caused by invasiveness, infection, and autoimmunity.

## 5. Other Hydrogel Products for Dry Eye

### 5.1. Lacrimal Plug

Mild dry eye can often be relieved through the use of lubricating eye drops. However, for patients with moderate and severe dry eye, a lacrimal plug is used to block lacrimal canaliculi to prolong lacrimal retention for long-term treatment [144]. Xu et al. [145] prepared a PVA/KGM/SA composite (PKS-2) hydrogel from konjac glucomannan (KGM), sodium alginate (SA), and PVA by calcium hydroxide crosslinking, which can be used as a potential material for lacrimal duct obstruction. In a rabbit dry eye model, the PKS-2 hydrogel was implanted into the rabbit eye as a plug. As expected, the hydrogel could prolong the tear retention time and slow down the tear excretion rate for 30 days.

### 5.2. Implants

Titanium implants are feasible choices for the repair of missing teeth because of their excellent biocompatibility and stability. The implant-mediated drug delivery system (IMDDS) has recently been used to treat chronic systemic diseases via slow drug release [146]. Pilocarpine can stimulate the muscarinic receptors to contract the smooth muscles and increase exocrine gland secretion. Highly soluble picropine was loaded into gelatin hydrogels, which were administered via IMDDS to prolong the effect of the promotion of tear secretion for 5 days (30 mg) and 7.5 days (60 mg) [92].

### 5.3. Eye Patches

The eye patch can provide a closed environment and reduce airflow and evaporation on the eye surface to preserve tears. Pang et al. [93] developed a photothermal conversion hydrogel-based mini-eye patch (GNRs@Pd hydrogel eye patch), which could change the eye patch temperature in accordance with the light intensity and then regulate the secretion of tears. The GNRs@Pd hydrogel patch was prepared with gold nanoparticles (GNRs), palladium (Pd) shell, gelatin (as raw materials), genipin (as the crosslinking agent), and temperature-sensitive ink (which was implanted into the eye patch). When the GNRs@Pd hydrogel patch was applied to the lacrimal gland, it could spontaneously sense a variety of visible light and heat intensities, stimulating the lacrimal gland to produce more tears so as to relieve dry eye symptoms. Interestingly, the color of the temperature-sensitive ink in the eye mask changed at different temperatures, which played an essential role in warning people against overusing their eyes. Significantly, the patch did not interfere with regular eye use.

## 6. Conclusions

This paper reviews the potential of hydrogels for the diagnosis and treatment of dry eye disease. The biosensors based on the hydrogel system proposed in this review can be used to detect different tear biomarkers, such as high-electric-point proteins and electrolytes, by virtue of other biosensors encapsulated in the hydrogels. Their minimally invasive nature prevents changes in the tear proteomes, electrolytes, and metabolites caused by external stimulation from affecting the diagnostic results. Biosensors based on hydrogel systems have several advantages, including their minimal invasiveness and capacity for the continuous monitoring of biomarkers. Because of this technique’s low cost and simple operation, it is an affordable method that is promising for the detection of dry eye biomarkers. However, diagnostic methods with a high specificity, high sensitivity, good repeatability, and the possibility of integrating readout circuits are also highly demanded. Moreover, hydrogels offer several advantages in the context of ophthalmology, such as their high biocompatibility, prolonged activity, and the retention time of the active agents. Hydrogels in form of eye drops, contact lenses, and lacrimal duct plugs are beneficial for controlling the release and prolonging the retention time of the loaded drugs. The combination of hydrogels with advanced drug delivery systems, such as liposomes, nanoparticles, and inclusion complexes, is expected to further enhance the sustained and controlled drug release. In addition, several problems related to sterilization, the shelf life, and initial burst release must be overcome before hydrogel therapy can be used in patients.

## Figures and Tables

**Table 1:** Sensitivity and Specificity of Objective Clinical Signs of Dry Eye Disease. Reprinted with permission from Ref. [9]. Copyright 2011, Elsevier.

Test	Cutoff	Sensitivity (*n* = 224)	Specificity (*n* = 75)
Osmolarity	>311 mOsms/L	72.8%	92.0%
TBUT	<10 s	84.4%	45.3%
Schirmer	<18 mm	79.5%	50.7%
Corneal stain	>Grade 1	54.0%	89.3%
Conjunctival stain	>Grade 2	60.3%	90.7%
Meibomian grade	>Grade 5	61.2%	78.7%

**Table 2:** Recent advances in the diagnosis of the biomarkers of dry eye with hydrogels.

Hydrogel Material/Type	Biosensor	Biomarkers	Ref.
P(NIPAM-co-MAA)-based hydrogel	Anionic hydrogel-coated gold nanoshells (AuNSs)	High-isoelectric-point proteins (especially lysozyme and lactoferrin)	[14]
Ionic poly(nisopropylacrylamide-co-methacrylic acid) (PNM) hydrogels	AuNSs	Lysozyme and lactoferrin	[57]
Modern silicone hydrogel (SiHG)	Three sodium-sensitive fluorescent groups (SG-C16, SG-LPE, and SG-PL)	Na^+^	[62]
Commercial silica gel hydrogel (SiHG)	Na^+^- and Cl^−^-sensitive fluorescent groups	Na^+^ and Cl^−^	[61]
Modern silicone hydrogel	6HQ-C18 probe (pH), SPQ probe (Cl^−^)	pH (H_3_O^+^/OH^−^) and Cl^−^	[13]
Conventional commercial hydrogel	Adjustable sensitivity sensor readout circuit, tear sensor, and antenna	Tear evaporation	[45]

**Table 3:** Recent patents on hydrogel products for the treatment of DED.

Patent Number	Hydrogel Material/Type	Payload	Mode of Application	Indication	Advantage
WO2022015940-A1, US2022183969-A1	Multi-arm polyethylene glycol (PEG)	Dexamethasone	Insert	DED such as episodic flares	Sustained release and biodegradable
US2022087932-A1	Multi-arm PEG	CsA	Insert	DED	Provides an effective release over an extended period
WO2022066891-A1	Poly(ethylene glycol) succinimidyl glutarate (PEG-SG)	/	Intracanalicular insert	DED, blepharitis, or allergic conjunctivitis	Sustained release and biodegradable
US2022313615-A1	Hydroxyethyl methacrylate	A moisturizing-active constituent	CL	Myopia, dry eye	Releasing the moisturizing active constituent slowly over time
US2022331157-A1	PNIPAAm and butyl acrylate	/	Plug	DED	Increasing tear volume, improving punctal stenosis,
CN114191378-A	Guar gum, PVA, and boric acid	Diquafosol sodium	Instillation	DED	shear thinning, and self-healing properties, sustained release of diquafosol sodium

**Table 4:** Hydrogel products for treating DED that are on the market and under clinical trials.

Hydrogel Product	Company	Main Constituent	Indication	Dose/Dosage	Status
Hylo^®^ Gel	URSAPHARM, Saarbrücken, Germany	2 mg/mL of hyaluronic acid sodium salt, a citrate buffer, sorbitol, and water	Dry eye syndromes	1 drop per eye, twice a day	On market
Vidisic^®^ gel	Bausch & Lomb, Rochester, NY, USA	Carbomer (CARBOPOL 980 NF)	DED	1 drop each time, at bedtime	On market
GelTears^®^	Bausch & Lomb	Carbomer 980	Moderate-severe DED		On market
Viscotears^®^	Novartis, Basel, Switzerland	2 mg/g Carbomer 980	Dry Eyes	3 or 4 × 1 drop per day	On market
Clinitas Gel^®^	Altacor, Reading, UK	Carbomer 980	Dry eye syndrome	3 × 1 drop per day	In market
Vitamin A Palmitate Eye Gel	Shenyang Xingqi Pharmaceutical Co., Ltd., Shenyang, China	Vitamin A Palmitate	As an adjunct therapy for corneal protection: dry eye with various causes	3 × 1 drop per day	In market
Bovine basic fibroblast growth factor (bFGF) gel	Zhuhai Yisheng Biological Pharmaceutical Co., Ltd., Zhuhai, China	bFGF	Dry eye	Four times a day	Phase 4
KIO-201 (Ocular Bandage Gel) [68]	EyeGate, Salt Lake City, UT, USA	0.75% (CMHA-S)	Persistent corneal epithelial fefect	4 × 1 drop per day	Phase 2
VisuXL^®^ Gel [69]	VISUfarma SpA, Rome, Italy	Coenzyme Q10, Vitamin E TPGS and cross-linked sodium carboxymethylcellulose	Dry Eye Syndromes	1 drop per eye, twice a day	Clinical trial
THEALOZ DUO GEL	URSAPHARM (Saarbrücken, Germany)	Trehalose, Natriumhyaluronat, Carbomer	Dry eye disease Eye diseases Other disorders of the lacrimal gland	3 × 1 drop per day	Recruiting

**Table 5:** Recent advances in the treatment of dry eye with drug-loading hydrogels.

Hydrogel Material/Type	Payload	Mode of Application	Advantages	Ref.
HA-VS and HA-SH hydrogel	HA	Instillation	Remains on the eye surface for at least 5 h	[76]
Nanoscale PAAc-based hydrogels	PAAc	Instillation	Improves the signs of eye dryness more efficiently and rapidly (3 days)	[77]
Gelatin, poly(*n*-isopropyl acrylamide), and lectin Helix pomatia agglutinin	Epigallocatechin gallate (EGCG)	Instillation	Retention time of up to 14 days	[82]
MPOSS-PEG-PPG (MPEP) thermogel copolymer	FK506	Instillation	Prolonged retention time of FK506 on the ocular surface	[85]
Carbopol 980	Phospho-sulindac (PS)	Instillation	Extended retention time when applied once a day	[88,89]
Dex-SA supramolecular hydrogel	Dexamethone, Dex-SA	Instillation	Nanofiber structure and thixotropy, prolonged anterior corneal retention time	[90]
Poly(ɛ-caprolactone)-poly(ethylene glycol)-poly(ɛ-caprolactone) copolymer (PCEC)	Pimecrolimus	Instillation	Sustained release of pimecrolimus	[86]
PVA/KGM/SA composite (PKS-2) hydrogel	/	Implant	Slows down the excretion of tears and lasts for 30 days	[91]
Hydroxybutyl chitosan (HBC)	/	Intracapsular injection (implant)	“Liquid plug”	[87]
Gelatin hydrogel	Pilocarpine	Implant	Prolonged effect of promoting tear secretion for 5 days (30 Mg) and 7.5 days	[92]
GNRs @ Pd hydrogel eye patch	/	Patch	Regulates the secretion of tears in accordance with the intensity of light	[93]
Silicone hydrogel	HA	CL	Improved hydrophilicity and wettability	[94]
pHEMA/TRIS silicone hydrogels	Crosslinked HA PRG4	CL	Reduced friction	[95]
Silicone hydrogel	HA-physisorbed/rhPRG4-grafted sample	CL	Surface wettability, antifouling, and water retention properties	[96]
PHEMA hydrogel	Thiolated HA	CL	Reduced contact angle, dehydration rate, and non-specific adsorption of lysozyme and albumin	[97]
p-HEMA hydrogel	HA	CL	Reduced contact angle and increased swelling rate	[98]
Cyclic PVA hydrogel	HA-loaded NPs	CL	HA release lasting for 14 days	[99]
Conventional hydrogel (consist of HEMA)	HA	CL	Releases HA for 10 days according to the treatment level	[100]
Silicon-copolymerized hydrogel contact	HPMC	CL	Regulates HPMC release by pH	[101]
p-HEMA-NIPAAm hydrogel, p-HEMA-VP hydrogel	HPMC	CL	Regulaties HPMC release by pH	[102]
HEMA hydrogel lenses	CsA	CL	CsA-eluting CLs release CsA for 48 h	[103]
pH-sensitive hydrogel (Eudragit S100)	CsA	CL	Sustained release of CsA for 14 days	[104]
PHEMA hydrogel	CsA	CL	Increased CsA bioavailability by at least 82%	[105]
Commercial silicone hydrogel lenses	Diquafosol	CL	Sustained release of diquafosol for 5 h	[106]
Non-ionic silicone hydrogel	Ap4A	CL	The release of Ap4A lasting for 300 min	[107]
Silicone hydrogel	Vitamin E, betaine, and dextran	CL	Loaded with 20–23% vitamin E to prolong the release time to 10 h	[108]
Silicon-based hydrogel	Betamethasone (BMZ) and Vitamin E	CL	Enhanced loading capacity of BMZ and reduced release rate of vitamin E	[109]
Silicone hydrogel	HPMC, trehalose, ibuprofen, and prednisolone	CL	Well-controlled release rate	[110]
Conventional hydrogel	Purified gastrin mucins	CL	Automatic lubrication, effective against dry eye	[111]

## Data Availability

Not applicable.

## References

[1] Shen Lee B., Toyos M., Karpecki P., Schiffbauer J., Sheppard J. (2022). Selective Pharmacologic Therapies for Dry Eye Disease Treatment: Efficacy, Tolerability, and Safety Data Review from Preclinical Studies and Pivotal Trials. Ophthalmol. Ther..

[2] Tan L.L., Morgan P., Cai Z.Q., Straughan R.A. (2015). Prevalence of and risk factors for symptomatic dry eye disease in Singapore. Clin. Exp. Optom..

[3] Luo Y., Yang W., Qi M., Wang Y., Li S., Wang M., Zeng Q. (2021). Annual direct economic burden and influencing factors of dry eye disease in Central China. Ophthalmic Epidemiol..

[4] Barrientos R.T., Godin F., Rocha-De-Lossada C., Soifer M., Sanchez-Gonzalez J.M., Moreno-Toral E., Gonzalez A.L., Zein M., Larco P.J., Mercado C. (2022). Ophthalmological Approach for the Diagnosis of Dry Eye Disease in Patients with Sjogren’s Syndrome. Life.

[5] Messmer E.M. (2015). The pathophysiology, diagnosis, and treatment of dry eye disease. Dtsch. Arztebl. Int..

[6] Lin P.H., Jian H.J., Li Y.J., Huang Y.F., Anand A., Huang C.C., Lin H.J., Lai J.Y. (2022). Alleviation of dry eye syndrome with one dose of antioxidant, anti-inflammatory, and mucoadhesive lysine-carbonized nanogels. Acta Biomater..

[7] Gupta P.K., Asbell P., Sheppard J. (2020). Current and Future Pharmacological Therapies for the Management of Dry Eye. Eye Contact Lens: Sci. Clin. Pract..

[8] Perez V.L., Stern M.E., Pflugfelder S.C. (2020). Inflammatory basis for dry eye disease flares. Exp. Eye Res..

[9] Lemp M.A., Bron A.J., Baudouin C., Benítez Del Castillo J.M., Geffen D., Tauber J., Foulks G.N., Pepose J.S., Sullivan B.D. (2011). Tear Osmolarity in the Diagnosis and Management of Dry Eye Disease. Am. J. Ophthalmol..

[10] Bron A.J., Willshire C. (2021). Tear Osmolarity in the Diagnosis of Systemic Dehydration and Dry Eye Disease. Diagnostics.

[11] Asbell P.A., Maguire M.G., Peskin E., Bunya V.Y., Kuklinski E.J. (2018). Dry Eye Assessment and Management (DREAM(c)) Study: Study design and baseline characteristics. Contemp. Clin. Trials..

[12] Girard B., de Saint S.G. (2021). Tear osmolarity, dry eye syndrome, blepharospasm and botulinum neurotoxin. J. Fr. Ophtalmol..

[13] Badugu R., Jeng B.H., Reece E.A., Lakowicz J.R. (2018). Contact lens to measure individual ion concentrations in tears and applications to dryeye disease. Anal. Biochem..

[14] Wechsler M.E., Dang H., Simmonds S.P., Bahrami K., Wyse J.M., Dahlhauser S.D., Reuther J.F., VandeWalle A.N., Anslyn E.V., Peppas N.A. (2021). Electrostatic and Covalent Assemblies of Anionic Hydrogel-Coated Gold Nanoshells for Detection of DryEye Biomarkers in Human Tears. Nano Lett..

[15] Azkargorta M., Soria J., Acera A., Iloro I., Elortza F. (2017). Human tear proteomics and peptidomics in ophthalmology: Toward the translation of proteomic biomarkers into clinical practice. J. Proteom..

[16] Lee J.D., Kim H.Y., Park J.J., Oh S.B., Goo H., Cho K.J., Kim S., Kim K.B. (2021). Metabolomics approach to biomarkers of dry eye disease using (1)H-NMR in rats. J. Toxicol. Environ. Health Part A.

[17] Zhang Y., Li J.M., Lu R., Liu Z., Chen X., de Paiva C.S., Pflugfelder S.C., Li D.Q. (2022). Imbalanced IL-37/TNF-alpha/CTSS signaling disrupts corneal epithelial barrier in a dry eye model in vitro. Ocul. Surf..

[18] Roda M., Corazza I., Bacchi R.M., Pellegrini M., Taroni L., Giannaccare G., Versura P. (2020). Dry Eye Disease and Tear Cytokine Levels-A Meta-Analysis. Int. J. Mol. Sci..

[19] Kook K.Y., Jin R., Li L., Yoon H.J., Yoon K.C. (2020). Tear Osmolarity and Matrix Metallopeptidase-9 in Dry Eye Associated with Sjogren’s Syndrome. Korean J. Ophthalmol..

[20] Fong P.Y., Shih K.C., Lam P.Y., Chan T.C.Y., Jhanji V., Tong L. (2019). Role of tear film biomarkers in the diagnosis and management of dry eye disease. Taiwan J. Ophthalmol..

[21] Ma B., Zhou Y., Liu R., Zhang K., Yang T., Hu C., Gao Y., Lan Q., Liu Y., Yang X. (2021). Pigment epithelium-derived factor (PEDF) plays anti-inflammatory roles in the pathogenesis of dry eye disease. Ocul. Surf..

[22] Jongkhajornpong P., Anothaisintawee T., Lekhanont K., Numthavaj P., McKay G., Attia J., Thakkinstian A. (2021). Short-term Efficacy and Safety of Biological Tear Substitutes and Topical Secretagogues for Dry Eye Disease: A Systematic Review and Network Meta-analysis. Cornea.

[23] Chen X., Wu J., Lin X., Wu X., Yu X., Wang B., Xu W. (2022). Tacrolimus Loaded Cationic Liposomes for Dry Eye Treatment. Front. Pharmacol..

[24] Muller L., Jensen B.P., Bachmann L.M., Wong D., Wells A.P. (2020). New technique to reduce systemic side effects of timolol eye drops: The tissue press method-Cross-over clinical trial. Clin. Exp. Ophthalmol..

[25] Zhang L., Su J.Z., Cai Z.G., Lv L., Zou L.H., Liu X.J., Wu J., Zhu Z.H., Mao C., Wang Y. (2019). Factors influencing the long-term results of autologous microvascular submandibular gland transplantation for severe dry eye disease. Int. J. Oral Maxillofac. Surg..

[26] Zhang X.D., Wei D.Y., Xu Y., Zhu Q. (2021). Hyaluronic acid in ocular drug delivery. Carbohydr. Polym..

[27] Park S.Y., Kang J.H., Kim H.S., Hwang J.Y., Shin U.S. (2022). Electrical and thermal stimulus-responsive nanocarbon-based 3D hydrogel sponge for switchable drug delivery. Nanoscale.

[28] Rajput A., Kulkarni M., Deshmukh P., Pingale P., Garkal A., Gandhi S., Butani S. (2021). A key role by polymers in microneedle technology: A new era. Drug Dev. Ind. Pharm..

[29] Bao Z., Yu A., Shi H., Hu Y., Jin B., Lin D., Dai M., Lei L., Li X., Wang Y. (2021). Glycol chitosan/oxidized hyaluronic acid hydrogel film for topical ocular delivery of dexamethasone and levofloxacin. Int. J. Biol. Macromol..

[30] Alruwaili N.K., Zafar A., Imam S.S., Alharbi K.S., Alotaibi N.H., Alshehri S., Alhakamy N.A., Alzarea A.I., Afzal M., Elmowafy M. (2020). Stimulus Responsive Ocular Gentamycin-Ferrying Chitosan Nanoparticles Hydrogel: Formulation Optimization, Ocular Safety and Antibacterial Assessment. Int. J. Nanomed..

[31] You Y., Xie Y., Jiang Z. (2019). Injectable and biocompatible chitosan-alginic acid hydrogels. Biomed. Mater..

[32] Yadav I., Purohit S.D., Singh H., Das N., Roy P., Mishra N.C. (2021). A highly transparent tri-polymer complexin situhydrogel of HA, collagen and four-arm-PEG as potential vitreous substitute. Biomed. Mater..

[33] Chan P.S., Xian J.W., Li Q., Chan C.W., Leung S., To K. (2019). Biodegradable Thermosensitive PLGA-PEG-PLGA Polymer for Non-irritating and Sustained Ophthalmic Drug Delivery. AAPS J..

[34] Jiang Y., Wang Y., Li Q., Yu C., Chu W. (2020). Natural Polymer-based Stimuli-responsive Hydrogels. Curr. Med. Chem..

[35] Hasan M., Zafar A., Yousaf M., Gulzar H., Mehmood K., Hassan S.G., Saeed A., Yousaf A., Mazher A., Rongji D. (2019). Synthesis of Loureirin B-Loaded Nanoliposomes for Pharmacokinetics in Rat Plasma. ACS Omega.

[36] Jacob S., Nair A.B., Shah J., Gupta S., Boddu S.H.S., Sreeharsha N., Joseph A., Shinu P., Morsy M.A. (2022). Lipid Nanoparticles as a Promising Drug Delivery Carrier for Topical Ocular Therapy—An Overview on Recent Advances. Pharmaceutics.

[37] Rebers L., Reichsollner R., Regett S., Tovar G., Borchers K., Baudis S., Southan A. (2021). Differentiation of physical and chemical cross-linking in gelatin methacryloyl hydrogels. Sci. Rep..

[38] Han Y., Cao Y., Lei H. (2022). Dynamic Covalent Hydrogels: Strong yet Dynamic. Gels.

[39] Li J.Y., Feng Y.H., He Y.T., Hu L.F., Liang L., Zhao Z.Q., Chen B.Z., Guo X.D. (2022). Thermosensitive hydrogel microneedles for controlled transdermal drug delivery. Acta Biomater..

[40] Shitrit Y., Bianco-Peled H. (2021). Insights into the formation mechanisms and properties of pectin hydrogel physically cross-linked with chitosan nanogels. Carbohydr. Polym..

[41] Torres-Luna C., Fan X., Domszy R., Hu N., Wang N.S., Yang A. (2020). Hydrogel-based ocular drug delivery systems for hydrophobic drugs. Eur. J. Pharm. Sci..

[42] Li H., Zhu X., Wang M., Zhao D., Li H., Yan J. (2022). Drug sustained release from degradable drug-loaded in-situ hydrogels in the posterior eye: A mechanistic model and analytical method. J. Biomech..

[43] Shirzaei S.E., Kheirkhah A., Rana D., Sun Z., Foulsham W., Sheikhi A., Khademhosseini A., Dana R., Annabi N. (2019). Sutureless repair of corneal injuries using naturally derived bioadhesive hydrogels. Sci. Adv..

[44] Santhanam S., Shui Y.B., Struckhoff J., Karakocak B.B., Hamilton P.D., Harocopos G.J., Ravi N. (2019). Bioinspired Fibrillary Hydrogel with Controlled Swelling Behavior: Applicability as an Artificial Vitreous. ACS Appl. Bio. Mater..

[45] Chiou J.C., Hsu S.H., Huang Y.C., Yeh G.T., Dai K.S., Kuei C.K. Towards a Fully Integrated, Wirelessly Powered, and Ordinarily Equipped On-lens System for Successive DryEye Syndrome Diagnosis. Proceedings of the 2017 Symposium on VLSI Technology.

[46] Zhang Z., Ma X., Xia T., Wu Y., Shi S., Lei L., Li X., Chen H., Lin D. (2021). A Novel Indomethacin-Tripeptide Hydrogel for Inhibiting Ocular Inflammation. J. Biomed. Nanotechnol..

[47] Wu Y., Liu Y., Li X., Kebebe D., Zhang B., Ren J., Lu J., Li J., Du S., Liu Z. (2019). Research progress of in-situ gelling ophthalmic drug delivery system. Asian J. Pharm. Sci..

[48] Young G., Hall L., Sulley A., Osborn-Lorenz K., Wolffsohn J.S. (2017). Inter-relationship of Soft Contact Lens Diameter, Base Curve Radius, and Fit. Optom. Vis. Sci..

[49] Zidan G., Greene C.A., Etxabide A., Rupenthal I.D., Seyfoddin A. (2021). Gelatine-based drug-eluting bandage contact lenses: Effect of PEGDA concentration and manufacturing technique. Int. J. Pharm..

[50] Alvarez-Lorenzo C., Anguiano-Igea S., Varela-Garcia A., Vivero-Lopez M., Concheiro A. (2019). Bioinspired hydrogels for drug-eluting contact lenses. Acta Biomater..

[51] Torres-Luna C., Hu N., Tammareddy T., Domszy R., Yang J., Wang N.S., Yang A. (2019). Extended delivery of non-steroidal anti-inflammatory drugs through contact lenses loaded with Vitamin E and cationic surfactants. Contact Lens Anterior Eye.

[52] Kraaij S., de Visscher J., Apperloo R.C., Nazmi K., Bikker F.J., Brand H.S. (2022). Lactoferrin and the development of salivary stones: A pilot study. Biometals.

[53] Vagge A., Senni C., Bernabei F., Pellegrini M., Scorcia V., Traverso C.E., Giannaccare G. (2020). Therapeutic Effects of Lactoferrin in Ocular Diseases: From Dry Eye Disease to Infections. Int. J. Mol. Sci..

[54] Ballard Z., Bazargan S., Jung D., Sathianathan S., Clemens A., Shir D., Al-Hashimi S., Ozcan A. (2020). Contact lens-based lysozyme detection in tear using a mobile sensor. Lab Chip..

[55] Berra M., Galperin G., Berra F., Marquez M.I., Mandaradoni M., Tau J., Berra A. (2021). Tear Lysozyme in Sjogren s syndrome, Meibomian gland dysfunction, and non-dry-eye. Arq. Bras. Oftalmol..

[56] Doctor M.B., Basu S. (2022). Lacrimal Gland Insufficiency in Aqueous Deficiency Dry Eye Disease: Recent Advances in Pathogenesis, Diagnosis, and Treatment. Seminars in Ophthalmology.

[57] Culver H.R., Wechsler M.E., Peppas N.A. (2018). Label-Free Detection of Tear Biomarkers Using Hydrogel-Coated Gold Nanoshells in a Localized Surface Plasmon Resonance-Based Biosensor. ACS Nano.

[58] Masoudi S. (2022). Biochemistry of human tear film: A review. Exp. Eye Res..

[59] von Thun Und Hohenstein-Blaul N., Funke S., Grus F.H. (2013). Tears as a source of biomarkers for ocular and systemic diseases. Exp. Eye Res..

[60] Tabernero J., Garcia-Porta N., Artal P., Pardhan S. (2021). Intraocular Scattering, Blinking Rate, and Tear Film Osmolarity After Exposure to Environmental Stress. Transl. Vis. Sci. Technol..

[61] Badugu R., Szmacinski H., Reece E.A., Jeng B.H., Lakowicz J.R. (2020). Fluorescent contact lens for continuous non-invasive measurements of sodium and chloride ion concentrations in tears. Anal. Biochem..

[62] Badugu R., Szmacinski H., Reece E.A., Jeng B.H., Lakowicz J.R. (2021). Sodium-sensitive contact lens for diagnostics of ocular pathologies. Sens Actuators B Chem..

[63] Mirzajani H., Mirlou F., Istif E., Singh R., Beker L. (2022). Powering smart contact lenses for continuous health monitoring: Recent advancements and future challenges. Biosens. Bioelectron..

[64] Pucker A.D. (2020). A Review of the Compatibility of Topical Artificial Tears and Rewetting Drops with Contact Lenses. Cont. Lens Anterior Eye.

[65] Labetoulle M., Benitez-del-Castillo J.M., Barabino S., Herrero Vanrell R., Daull P., Garrigue J., Rolando M. (2022). Artificial Tears: Biological Role of Their Ingredients in the Management of Dry Eye Disease. Int. J. Mol. Sci..

[66] Arshinoff S.A., Hofmann I., Nae H. (2021). Role of rheology in tears and artificial tears. J. Cataract Refract. Surg..

[67] Yazdanpanah G., Jiang Y., Rabiee B., Omidi M., Rosenblatt M.I., Shokuhfar T., Pan Y., Naba A., Djalilian A.R. (2021). Fabrication, Rheological, and Compositional Characterization of Thermoresponsive Hydrogel from Cornea. Tissue Eng. Part C Methods.

[68] Wolsey D., Slade S., Wirostko B.M., Brandano L.A., Mann B.K., Durrie D.S., Thompson V. (2020). Novel Cross-Linked Ocular Bandage Gel Improves Reepithelialization After Photorefractive Keratectomy: A Randomized, Masked Prospective Study. J. Ocul. Pharmacol. Ther..

[69] Tredici C., Fasciani R., Villano A., Gambini G., Caporossi A. (2020). Efficacy of eye drops containing crosslinked hyaluronic acid and CoQ10 in restoring ocular health exposed to chlorinated water. Eur. J. Ophthalmol..

[70] Araújo D.M.L.D., Galera P.D. (2016). Ocular lubricants: What is the best choice?. Ciência. Rural..

[71] Guter M., Breunig M. (2017). Hyaluronan as a promising excipient for ocular drug delivery. Eur. J. Pharm. Biopharm..

[72] Fallacara A., Baldini E., Manfredini S., Vertuani S. (2018). Hyaluronic Acid in the Third Millennium. Polymers.

[73] Posarelli C., Passani A., Del Re M., Fogli S., Toro M.D., Ferreras A., Figus M. (2019). Cross-Linked Hyaluronic Acid as Tear Film Substitute. J. Ocul. Pharmacol. Ther..

[74] Williams D.L., Mann B.K. (2014). Efficacy of a Crosslinked Hyaluronic Acid-Based Hydrogel as a Tear Film Supplement: A Masked Controlled Study. PLoS ONE.

[75] Williams D.L., Mann B.K. (2013). A Crosslinked HA-Based Hydrogel Ameliorates Dry Eye Symptoms in Dogs. Int. J. Biomater..

[76] Yu Y., Chow D., Lau C., Zhou G.Q., Back W., Xu J., Carim S., Chau Y. (2021). A bioinspired synthetic soft hydrogel for the treatment of dryeye. Bioeng Transl Med..

[77] Swilem A.E., Elshazly A., Hamed A.A., Hegazy E.A., Abd E.H. (2020). Nanoscale poly(acrylic acid)-based hydrogels prepared via a green single-step approach for application as low-viscosity biomimetic fluid tears. Mater. Sci. Eng. C Mater. Biol. Appl..

[78] Qiu Y., Park K. (2001). Environment-sensitive hydrogels for drug delivery. Adv. Drug Deliv. Rev..

[79] Yu Y., Cheng Y., Tong J., Zhang L., Wei Y., Tian M. (2021). Recent advances in thermo-sensitive hydrogels for drug delivery. J. Mater. Chem. B.

[80] Xian S., Webber M.J. (2020). Temperature-responsive supramolecular hydrogels. J. Mater. Chem. B.

[81] Xu L., Liang X., You L., Yang Y., Fen G., Gao Y., Cui X. (2021). Temperature-sensitive poly(N-isopropylacrylamide)-chitosan hydrogel for fluorescence sensors in living cells and its antibacterial application. Int. J. Biol. Macromol..

[82] Luo L.J., Nguyen D.D., Lai J.Y. (2020). Long-acting mucoadhesive thermogels for improving topical treatments of dryeye disease. Mater. Sci. Eng. C Mater. Biol. Appl..

[83] Wu Y., Yao J., Zhou J., Dahmani F.Z. (2013). Enhanced and sustained topical ocular delivery of cyclosporine A in thermosensitive hyaluronic acid-based in situ forming microgels. Int. J. Nanomed..

[84] Schutte-Nutgen K., Tholking G., Suwelack B., Reuter S. (2018). Tacrolimus—Pharmacokinetic Considerations for Clinicians. Curr. Drug Metab..

[85] Han Y., Jiang L., Shi H.H., Xu C.F., Liu M.T., Li Q.J., Zheng L., Chi H., Wang M.Y., Liu Z.G. (2022). Effectiveness of an ocular adhesive polyhedral oligomeric silsesquioxane hybrid thermo-responsive FK506 hydrogel in a murine model of dry eye. Bioact. Mater..

[86] Fan Y.F., Zhuang B., Wang C., Xu X.L., Xu W., Lv Z.H. (2016). Pimecrolimus micelle exhibits excellent therapeutic effect for Keratoconjunctivitis Sicca. Colloids Surf. B Biointerfaces.

[87] Lin T., Lu Y., Zhang X.Z., Gong L., Wei C.Z. (2018). Treatment of dry eye by intracanalicular injection of a thermosensitive chitosan-based hydrogel: Evaluation of biosafety and availability. Biomater. Sci..

[88] Huang W., Huang L.Q., Li W.Y., Saglam M.S., Tourmouzis K., Goldstein S.M., Master A., Honkanen R., Rigas B. (2022). Once-Daily Topical Phosphosulindac Is Efficacious in the Treatment of DryEye Disease: Studies in Rabbit Models of Its Main Clinical Subtypes. J. Ocul. Pharmacol. Ther..

[89] Huang W., Huang L.Q., Tsioulias A., Wen Z.Y., Saglam S., Goldstein S.M., Honkanen R., Rigas B. (2022). Hydrogel formulation of phosphosulindac allows once-a-day ocular dosing and limits its biodistribution to the anterior chamber: Application to dryeye disease treatment. J. Drug Deliv. Sci. Technol..

[90] Zhang Z.L., Yu J., Zhou Y.F., Zhang R.S., Song Q.Q., Lei L., Li X.Y. (2018). Supramolecular nanofibers of dexamethasone derivatives to form hydrogel for topical ocular drug delivery. Colloids Surf. B Biointerfaces.

[91] Xu N.X., Yang H., Wei R., Pan S.Y., Huang S.P., Xiao X.H., Wen H.Y., Xue W.M. (2019). Fabrication of Konjac glucomannan-based composite hydrogel crosslinked by calcium hydroxide for promising lacrimal plugging purpose. Int. J. Biol. Macromol..

[92] Cha S., Kim H.K., Kho H.S., Park Y.S. (2017). The Sustained Effects on Tear Volume of Pilocarpine Hydrochloride in Gelatin by Hydrogel Administered by An Implant-mediated Drug Delivery System. Curr. Drug Deliv..

[93] Pang Y.L., Wei C.C., Li R.L., Wu Y., Liu W., Wang F.F., Zhang X., Wang X.L. (2019). Photothermal conversion hydrogel based mini-eye patch for relieving dryeye with long-term use of the light-emitting screen. Int. J. Nanomed..

[94] Yamasaki K., Drolle E., Nakagawa H., Hisamura R., Ngo W., Jones L. (2021). Impact of a low molecular weight hyaluronic acid derivative on contact lens wettability. Contact Lens Anterior Eye.

[95] Samsom M., Korogiannaki M., Subbaraman L.N., Sheardown H., Schmidt T.A. (2018). Hyaluronan incorporation into model contact lens hydrogels as a built-in lubricant: Effect of hydrogel composition and proteoglycan 4 as a lubricant in solution. J. Biomed. Mater. Res. B Appl. Biomater..

[96] Korogiannaki M., Samsom M., Matheson A., Soliman K., Schmidt T.A., Sheardown H. (2021). Investigating the Synergistic Interactions of Surface Immobilized and Free Natural Ocular Lubricants for Contact Lens Applications: A Comparative Study between Hyaluronic Acid and Proteoglycan 4 (Lubricin). Langmuir.

[97] Korogiannaki M., Zhang J.F., Sheardown H. (2017). Surface modification of model hydrogel contact lenses with hyaluronic acid via thiol-ene “click” chemistry for enhancing surface characteristics. J. Biomater. Appl..

[98] Maulvi F.A., Soni T.G., Shah D.O. (2015). Extended release of hyaluronic acid from hydrogel contact lenses for dryeye syndrome. J. Biomater. Sci. Polym. Ed..

[99] Akbari E., Imani R., Shokrollahi P., Keshel S.H. (2021). Preparation of Nanoparticle-Containing Ring-Implanted Poly(Vinyl Alcohol) Contact Lens for Sustained Release of Hyaluronic Acid. Macromol. Biosci..

[100] Maulvi F.A., Shetty K.H., Desai D.T., Shah D.O., Willcox M. (2021). Recent advances in ophthalmic preparations: Ocular barriers, dosage forms and routes of administration. Int. J. Pharm..

[101] Kim G., Kim H.J., Noh H. (2019). Influence of Solution pH on Drug Release from Ionic Hydrogel Lens. Macromol. Res..

[102] Kim G., Kim H.J., Noh H. (2018). pH Sensitive Soft Contact Lens for Selective Drug-Delivery. Macromol. Res..

[103] Choi J.H., Li Y., Jin R., Shrestha T., Choi J.S., Lee W.J., Moon M.J., Ju H.T., Choi W., Yoon K.C. (2019). The Efficiency of Cyclosporine A-Eluting Contact Lenses for the Treatment of Dry Eye. Curr. Eye Res..

[104] Maulvi F.A., Choksi H.H., Desai A.R., Patel A.S., Ranch K.M., Vyas B.A., Shah D.O. (2017). pH triggered controlled drug delivery from contact lenses: Addressing the challenges of drug leaching during sterilization and storage. Colloids Surf B Biointerfaces.

[105] Qin R., Guo Y., Ren H., Liu Y., Su H., Chu X., Jin Y., Lu F., Wang B., Yang P. (2022). Instant Adhesion of Amyloid-like Nanofilms with Wet Surfaces. ACS Cent. Sci..

[106] Dominguez-Godinez C., Carracedo G., Pintor J. (2018). Diquafosol Delivery from Silicone Hydrogel Contact Lenses: Improved Effect on Tear Secretion. J. Ocul. Pharmacol. Ther..

[107] Dominguez-Godinez C.O., Martin-Gil A., Carracedo G., Guzman-Aranguez A., González-Méijome J.M., Pintor J. (2013). In vitro and in vivo delivery of the secretagogue diadenosine tetraphosphate from conventional and silicone hydrogel soft contact lenses. J. Optom..

[108] Hsu K.H., de la Jara P.L., Ariyavidana A., Watling J., Holden B., Garrett Q., Chauhan A. (2015). Release of Betaine and Dexpanthenol from Vitamin E Modified Silicone-Hydrogel Contact Lenses. Curr. Eye Res..

[109] Rad M.S., Tabassi S., Moghadam M.H., Mohajeri S.A. (2016). Controlled release of betamethasone from vitamin E-loaded silicone-based soft contact lenses. Pharm. Dev. Technol..

[110] White C.J., DiPasquale S.A., Byrne M.E. (2016). Controlled Release of Multiple Therapeutics from Silicone Hydrogel Contact Lenses. Optom. Vis. Sci..

[111] Winkeljann B., Boettcher K., Balzer B.N., Lieleg O. (2017). Mucin Coatings Prevent Tissue Damage at the Cornea-Contact Lens Interface. Adv. Mater. Interfaces.

[112] Lee M.J., Park S.Y., Sung A.Y. (2021). Characterization of Biocompatible Hydrogel Lenses Using Methacrylic Acid with Neodymium Oxide Nanoparticles. Polymers.

[113] Tran N.P., Ting C.C., Lin C.H., Yang M.C. (2020). A Novel Approach to Increase the Oxygen Permeability of Soft Contact Lenses by Incorporating Silica Sol. Polymers.

[114] Kar A., Ahamad N., Dewani M., Awasthi L., Patil R., Banerjee R. (2022). Wearable and implantable devices for drug delivery: Applications and challenges. Biomaterials.

[115] Dixon P., Christopher K., Jovic N., Chauhan A. (2019). Spectroscopy of Oxygen-Sensitive Material for Measuring Contact Lens Oxygen Transmissibility. Curr. Eye Res..

[116] Hendi A., Umair H.M., Elsherif M., Alqattan B., Park S., Yetisen A.K., Butt H. (2020). Healthcare Applications of pH-Sensitive Hydrogel-Based Devices: A Review. Int. J. Nanomed..

[117] Hui A. (2017). Contact lenses for ophthalmic drug delivery. Clin. Exp. Optom..

[118] Creech J.L., Chauhan A., Radke C.J. (2001). Dispersive Mixing in the Posterior Tear Film Under a Soft Contact Lens. Ind. Eng. Chem. Res..

[119] Guzman-Aranguez A., Fonseca B., Carracedo G., Martin-Gil A., Martinez-Aguila A., Pintor J. (2016). Dry Eye Treatment Based on Contact Lens Drug Delivery: A Review. Eye Contact Lens..

[120] Chau T.N.D., Dowling J., Ryan R., McLoughlin P., Fitzhenry L. (2022). Controlled release of naringenin from soft hydrogel contact lens: An investigation into lens critical properties and in vitro release. Int. J. Pharm..

[121] Maulvi F.A., Soni T.G., Shah D.O. (2016). A review on therapeutic contact lenses for ocular drug delivery. Drug Deliv..

[122] Liu Z., Overton M., Chauhan A. (2022). Transport of Vitamin E from Ethanol/Water Solution into Contact Lenses and Impact on Drug Transport. J. Ocul. Pharmacol. Ther..

[123] DiPasquale S.A., Uricoli B., DiCerbo M.C., Brown T.L., Byrne M.E. (2021). Controlled Release of Multiple Therapeutics From Silicone Hydrogel Contact Lenses for Post-Cataract/Post-Refractive Surgery and Uveitis Treatment. Transl. Vis. Sci. Technol..

[124] Shin S.M., Park H.I., Sung A.Y. (2022). Development of Functional Ophthalmic Materials Using Natural Materials and Gold Nanoparticles. Micromachines.

[125] Lanier O.L., Christopher K.G., Macoon R.M., Yu Y., Sekar P., Chauhan A. (2020). Commercialization challenges for drug eluting contact lenses. Expert Opin. Drug Deliv..

[126] Catoira M.C., González-Payo J., Fusaro L., Ramella M., Boccafoschi F. (2020). Natural hydrogels R&D process: Technical and regulatory aspects for industrial implementation. J. Mater. Sci.: Mater. Med..

[127] Ono J., Toshida H. (2022). Use of Ketotifen Fumarate-Eluting Daily Disposable Soft Contact Lens in Management of Ocular Allergy: Literature Review and Report of Two Cases. Cureus.

[128] Jacobs D.S., Carrasquillo K.G., Cottrell P.D., Fernández-Velázquez F.J., Gil-Cazorla R., Jalbert I., Pucker A.D., Riccobono K., Robertson D.M., Szczotka-Flynn L. (2021). BCLA CLEAR—Medical use of contact lenses. Contact Lens Anterior Eye.

[129] Angeles G.H., Nesporova K. (2021). Hyaluronan and its derivatives for ophthalmology: Recent advances and future perspectives. Carbohydr. Polym..

[130] Weeks A., Morrison D., Alauzun J.G., Brook M.A., Jones L., Sheardown H. (2012). Photocrosslinkable hyaluronic acid as an internal wetting agent in model conventional and silicone hydrogel contact lenses. J. Biomed. Mater. Res. A.

[131] Weeks A., Luensmann D., Boone A., Jones L., Sheardown H. (2012). Hyaluronic acid as an internal wetting agent in model DMAA/TRIS contact lenses. J. Biomater. Appl..

[132] Paterson S.M., Liu L.N., Brook M.A., Sheardown H. (2015). Poly(ethylene glycol)-or silicone-modified hyaluronan for contact lens wetting agent applications. J. Biomed. Mater. Res. A.

[133] Weeks A., Boone A., Luensmann D., Jones L., Sheardown H. (2013). The effects of hyaluronic acid incorporated as a wetting agent on lysozyme denaturation in model contact lens materials. J. Biomater. Appl..

[134] Maulvi F.A., Shaikh A.A., Lakdawala D.H., Desai A.R., Pandya M.M., Singhania S.S., Vaidya R.J., Ranch K.M., Vyas B.A., Shah D.O. (2017). Design and optimization of a novel implantation technology in contact lenses for the treatment of dryeye syndrome: In vitro and in vivo evaluation. Acta Biomater..

[135] Ali M., Byrne M.E. (2009). Controlled Release of High Molecular Weight Hyaluronic Acid from Molecularly Imprinted Hydrogel Contact Lenses. Pharm. Res..

[136] White C.J., McBride M.K., Pate K.M., Tieppo A., Byrne M.E. (2011). Extended release of high molecular weight hydroxypropyl methylcellulose from molecularly imprinted, extended wear silicone hydrogel contact lenses. Biomaterials.

[137] Portal C., Gouyer V., Gottrand F., Desseyn J. (2019). Ocular mucins in dry eye disease. Exp. Eye Res..

[138] Kusama S., Sato K., Yoshida S., Nishizawa M. (2020). Self-Moisturizing Smart Contact Lens Employing Electroosmosis. Adv. Mater. Technol..

[139] Yamaguchi T. (2018). Inflammatory Response in Dry Eye. Invest. Ophthalmol. Vis. Sci..

[140] de Paiva C.S., Pflugfelder S.C., Ng S.M., Akpek E.K. (2019). Topical cyclosporine A therapy for dry eye syndrome Review. Cochrane Database Syst. Rev..

[141] Peng C.C., Chauhan A. (2011). Extended cyclosporine delivery by silicone-hydrogel contact lenses. J. Control Release.

[142] Chen W., Zhang X., Li J., Wang Y., Chen Q., Hou C., Garrett Q. (2013). Efficacy of osmoprotectants on prevention and treatment of murine dry eye. Invest. Ophthalmol. Vis. Sci..

[143] Li J.Y., Zhang X.H., Zheng Q.X., Zhu Y.R., Wang H., Ma H.X., Jhanji V., Chen W. (2015). Comparative Evaluation of Silicone Hydrogel Contact Lenses and Autologous Serum for Management of Sjogren Syndrome-Associated DryEye. Cornea.

[144] Dai M., Xu K., Xiao D., Zheng Y., Zheng Q., Shen J., Qian Y., Chen W. (2022). In Situ Forming Hydrogel as a Tracer and Degradable Lacrimal Plug for Dry Eye Treatment. Adv. Healthc Mater..

[145] Wen Z.Y., Muratomi N., Huang W., Huang L.Q., Ren J.F., Yang J., Persaud Y., Loloi J., Mallangada N., Kung P. (2019). The ocular pharmacokinetics and biodistribution of phospho-sulindac (OXT-328) formulated in nanoparticles: Enhanced and targeted tissue drug delivery. Int. J. Pharm..

[146] Park Y. (2017). Novel route of insulin delivery using an implant-mediated drug delivery system. Drug Deliv. Transl. Res..

